# Coronavirus disease 2019 and dengue: two case reports

**DOI:** 10.1186/s13256-021-02707-7

**Published:** 2021-03-26

**Authors:** Abdullah Isneen Hilmy, Rajib Kumar Dey, Hisham Ahmed Imad, Abdul Azeez Yoosuf, Ali Nazeem, Ali Abdulla Latheef

**Affiliations:** 1 Department of Internal Medicine, Indira Gandhi Memorial Hospital, Male’ 20002, Maldives; 2 Mahidol-Osaka Center for Infectious Diseases, Faculty of Tropical Medicine, Mahidol University, Bangkok 10400, Thailand; 3 Department of Viral Infections, Research Institute for Microbial Diseases, Osaka University, Osaka 565-0871, Japan; 4 Technical Advisory Group, Health Emergency Operation Center, Male’ 20002, Maldives

**Keywords:** COVID-19, Dengue, Coinfection, Pandemic, Case report

## Abstract

**Background:**

The pandemic of this century has overwhelmed the healthcare systems of affected countries, and all resources have been diverted to coronavirus disease 2019. At the onset, coronavirus disease 2019 can present as any other acute febrile undifferentiated illness. In tropical regions, clinicians are increasingly challenged to differentiate these febrile illnesses without the use of diagnostics. With this pandemic, many of these tropical diseases are neglected and go underreported. Dengue is holoendemic in the Maldives, and dengue viruses circulate throughout the year. Reports about coinfections with dengue virus and severe acute respiratory syndrome coronavirus 2 are scarce, and the outcome and the dynamics of the disease may be altered in the presence of coinfection. We have described the clinical manifestation and serial laboratory profile, and highlighted the atypical findings uncommon in dengue infection.

**Case presentation:**

Case 1 was a 39-year old Asian male, presented on day 6 of dengue infection with warning signs. Reverse transcription polymerase chain reaction for severe acute respiratory syndrome coronavirus 2 that was done as per hospital protocol was found to be positive. Case 2 was a 38-year old Asian male, was admitted on day 5 of illness with symptoms of acute respiratory infection with positive reverse transcription polymerase chain reaction for severe acute respiratory syndrome coronavirus 2. Evaluation of progressive leukopenia and thrombocytopenia showed positive dengue serology.

**Conclusion:**

Clinicians must be conscientious when working on the differential diagnosis of possible tropical diseases in cases of coronavirus disease 2019, specifically, when patients develop hemoconcentration, thrombocytopenia, and transaminitis with elevated expression of aspartate higher than alanine transaminase, which is frequently observed in dengue infection. Caution must be taken during the administration of intravenous fluids when treating patients with coronavirus disease 2019 and dengue coinfection, as coronavirus disease 2019 patients are more prone to develop pulmonary edema. Timely diagnosis and appropriate management are essential to avoid the devastating complications of severe forms of dengue infection. It is important to repeat and reconfirm the dengue serology in coronavirus disease 2019 patients to avoid false positivity. Diligence and care must be taken not to neglect other endemic tropical diseases in the region during the present pandemic.

## Introduction

The coronavirus disease 2019 (COVID-19) pandemic began in late December 2019. Over 19 million confirmed cases and 700,000 fatalities have been reported. At present, only 12 countries worldwide have not been affected by COVID-19 [1]. The first case of COVID-19 in the Maldives was confirmed on 7 March 2020 [2]. Most of the confirmed cases were asymptomatic, and among the symptomatic cases a majority had mild to moderate disease and a minority were critical. To date, the Maldives has had over 4769 confirmed cases of COVID-19 and 19 fatalities [3]. Several arboviral infections and other tropical diseases are endemic to the Maldives [4–13]. Among these, the most commonly diagnosed is dengue infection because of the widespread use of commercially available serological rapid diagnostic test kits [14]. Annually, the Maldives experiences a peak in the number of reported cases during the dengue outbreaks, especially during the monsoons. Factors contributing to this are poor vector control due to congested urbanization and limited efforts to segregate accumulated waste, which are potential vector breeding grounds [15]. As the COVID-19 pandemic has been the prime focus of attention in the past 7 months, many of the existing problems such as dengue have been neglected. Severe forms of dengue if left undetected can be fatal [16–18]. These include dengue hemorrhagic fever, where in addition to bleeding, plasma leakage occurs and expanded dengue syndrome with multisystemic involvement. These classifications are now collectively referred to as severe dengue [19]. Many countries where dengue is endemic have expressed concerns over the devastating impact a dengue outbreak could have during the COVID-19 pandemic [20–22], while others express how dengue and COVID-19 share similarities in both clinical and laboratory findings [23]. One report demonstrated a false dengue serological test in a COVID-19 case [24]. There are also reports of serological overlap resulting in false positive dengue serology [25]. Reports about coinfections with dengue virus and severe acute respiratory syndrome coronavirus 2 (SARS-CoV-2) are scarce [24, 26]. Herein, we describe the clinical manifestation and serial laboratory profile and highlight the atypical findings uncommon in dengue fever and dengue hemorrhagic fever when coinfected with SARS-CoV-2.

## Case presentation

### Case 1

A 39-year-old, Asian male, who is a migrant worker presented to the emergency department with a history of persistent high-grade fever, retro-orbital headache, fatigue, and myalgia for the past 6 days. He also complained of right upper quadrant pain, vomiting, and loose stools. There was no history of rash, arthralgia, or bleeding. On physical examination, he was dehydrated and the vitals recorded were a body temperature of 37.5°C, heart rate of 80 beats per minute, blood pressure of 113/78 mmHg, pulse pressure of 35 mmHg, respiratory rate of 20 breaths per minute, capillary refill time of less than 2 seconds, and oxygen saturation of 98% in room air. Systemic examination was unremarkable except for right upper quadrant tenderness with a normal liver span. Bedside ultrasonography of the abdomen revealed gall bladder wall edema. The laboratory investigations at presentation and serial laboratory profiles are tabulated in Table 1, which shows hemoconcentration of 19% and transaminitis. Dengue rapid test (SD BIOLINE Dengue DUO®) revealed a negative non-structural protein 1 (NS1) with a positive immunoglobulin (Ig)M and IgG. This was later reconfirmed with a positive dengue enzyme-linked immunosorbent assay (ELISA) IgM and IgG (Panbio^®^ Standard Diagnostics). Reverse transcription polymerase chain reaction (rRT-PCR) (Liferiver^TM^ 2019-nCoV Real Time Multiplex RT-PCR Kit) for COVID-19 done as per hospital admission protocol was positive with a cycle threshold of 28.35. The patient was admitted to a COVID-19 facility and kept in isolation with a diagnosis of dengue hemorrhagic fever grade I with COVID-19. As the patient was vitally stable with a minimal increase in hemoconcentration, he was managed conservatively with oral fluids. On day 5 of illness, the patient developed non-productive cough and sore throat for which a chest X-ray was done and no abnormality was found. The patient was monitored daily to look for development of warning signs of dengue fever, including hemoconcentration. The patient had an uneventful hospital stay and was discharged after completing the mandatory isolation period of 14 days.

**Table 1:** Serial laboratory parameters during the course of illness, Case 1

Day of illness	Day 6	Day 7	Day 9	Day 12	Day 15
Hemoglobin g/dL [range: 14–18]	15.5	16.3	16.10	14.80	14.80
Hematocrit % [range: 40–54]	46.90	45.1	44.70	43.80	39.70
Leukocytes 10^3^/µL [range: 5.0–10.0]	6.936	8.19	7.37	7.71	11.40
Neutrophils % [range: 45–74]	44.31	41.9	18.80	26.30	36.20
Lymphocytes % [range: 16–45]	42.80	42.3	65	57.10	47.40
Platelets 10^3^/µL [range: 150–450]	198.6	193	212	227	284
AST IU/L [range: 05–34]	100	101	68	56	
ALT IU/L [range: 00–55]	167	177	143	124	

*AST* aspartate transaminase, *ALT* alanine transaminase

### Case 2

A 38-year-old Asian male presented with a history of intermittent fever and generalized headache for the past 5 days. He also complained of sore throat, dysgeusia, and anosmia for a duration of 3 days following the onset of fever. Being a close contact of a COVID-19 patient, he consulted an online clinic where he was referred to the hospital for evaluation. On examination, he appeared dehydrated, with a body temperature of 37.4°C, pulse rate of 84 beats per minute, blood pressure of 100/60 mmHg, pulse pressure of 40 mmHg, respiratory rate of 21 breaths per minute, capillary refill time of less than 2 seconds, and oxygen saturation of 97% in room air. Other systemic examination was unremarkable. With a positive rRT-PCR for SARS-CoV-2 (Ct value 24.45) he was admitted and kept in isolation. His laboratory parameters are tabulated in Table 2, which shows leukopenia and thrombocytopenia. In view of the depleting trend of total leukocyte count and platelets, a dengue rapid test was requested, which showed a negative NS1 and positive IgM/IgG. This was reconfirmed with a positive dengue ELISA serology of anti-dengue IgM and IgG. Hence, the patient was diagnosed with COVID-19 with dengue fever. The patient had minimal hemoconcentration, and as he was able to take fluids adequately he was managed with oral fluids and monitored daily for the development of warning signs of dengue or worsening of the severity of COVID-19. The patient did not have any fever spikes after admission. The patient’s platelets gradually improved, and symptoms resolved over a period of 1 week. The patient was discharged after completing the 14-day mandatory period of isolation.

**Table 2:** Serial laboratory parameters during the course of illness, Case 2

Day of illness	Day 6	Day 7	Day 8	Day 11	Day 15
Hemoglobin g/dL [range: 14–18]	14.3	14.8	14.0	14.7	14.0
Hematocrit % [range: 40–54]	41.8	45.4	41.5	45.9	43.9
Leukocytes 10^3^/µL [range: 5.0–10.0]	3.14	2.81	3.27	4.45	8.08
Neutrophils % [range: 45–74]	28.5	14.7	15.6	31	41.9
Lymphocytes % [range: 16–45]	51.1	66.1	67.1	53.4	41.3
Platelets 10^3^/µL [range: 150–450]	158	156	136	159	236
AST IU/L [range: 05–34]	66		46		
ALT IU/L [range: 00–55]	44		48		

*AST* aspartate transaminase, *ALT* alanine transaminase

## Discussion

Here we describe two cases of coinfection of dengue fever and COVID-19. The first case had presented with symptoms of dengue fever, and after 5 days of admission he developed symptoms of COVID-19. The second case was admitted with mild COVID-19, and during the course of illness, in view of mild hemoconcentration and progressive decline in leukocyte and thrombocyte counts, he was tested positive for dengue fever. In coinfections, one virus can suppress or augment the other, leading to varying clinical manifestations of the diseases.

The COVID-19 pandemic has spread across the globe, including areas where other tropical diseases are endemic such as the Maldives [6, 8–10, 15]. Clinicians are challenged with the additional burden of possible coinfections with other tropical diseases that have the potential to complicate the course of illness [26]. Coinfections with COVID-19 have been reported with multiple bacteria and viruses [27–31]. Nevertheless, coinfection with dengue viruses and SARS-CoV-2 are still scarce, with only a few reports describing false-positive dengue serology [24, 32]. The healthcare system of the Maldives is stretched and may not respond adequately to a dual outbreak because of limited manpower and infrastructure [33]. Every year, the Maldives experiences a dengue outbreak during the monsoons. From the year 2011, an average of 1543 cases have been reported annually [15]. In 2018, 3494 cases were reported, and in 2019, 5023 cases were reported, which was a record number for the Maldives [34]. Despite an expected increase in cases, so far only 225 cases have been reported, while dengue cases are on the rise in other endemic regions [34, 35]. We postulate that healthcare providers are more focused on COVID-19, which has resulted in lower rates of testing or reporting of dengue virus. In addition, individuals with mild symptomatic dengue infections perhaps are not visiting hospitals because of the pandemic and lockdowns. COVID-19 may present as an undifferentiated acute febrile illness [36]. In one study, 87.9% of COVID-19 patients presented with fever, 67.7% presented with cough, and 13.7% of patients had headache [37]. Nevertheless, information regarding the frequency of fever in dengue infection is limited in most prospective studies, mostly due to confounders within the study designs where fever remains an inclusion criterion. Fever can be absent in the elderly population or, if present, can occur over 90% of the time [38]. Cough in dengue infection has been reported in 67% of cases, which can be due to pleural effusion observed during plasma leakage in dengue hemorrhagic fever (DHF) [39, 40]. Headache in dengue infection has been reported in 89% of cases and more commonly in DHF [41]. Bleeding in COVID-19 has been reported to be associated with morbidity, but frequency of bleeding manifestations in COVID-19 remains low [42]. In contrast, bleeding in dengue infection occurs more frequently and is associated with increasing severity [41]. The hematological profile in COVID-19 and dengue infection are similar. Neutrophils are increased early in both infections with leukopenia and thrombocytopenia. The discrepancy in the hematological profile in both infections is lymphopenia, which is observed in COVID-19 during the course of illness in contrast to a reverse neutrophil-to-lymphocyte ratio in dengue infection. Transaminitis is observed in both infections; however, the expression of aspartate aminotransferase is greater than alanine transaminase in dengue infection [43, 44]. Our first case was initially diagnosed as dengue fever with warning signs, and COVID-19 was detected during the screening process before admission [45]. On day 5 of admission, he developed a non-productive cough and sore throat that could be attributed to COVID-19. The cough could also be due to dengue-related pleural effusion, which was not evident on his chest X-ray in posterior–anterior view. However, a chest X-ray in lateral decubitus view is more sensitive to detect minimal effusion [46]. In our second case, the patient was admitted with a diagnosis of mild COVID-19. With a depleting trend of leukocytes and platelets, dengue serology was performed, which was positive. Case 1 had a hemoconcentration of 19% and gall bladder wall edema suggestive of plasma leakage [47]. As there was no bleeding manifestation, this case was classified as DHF grade 1 [48]. In case 1, liver biochemistry revealed an atypical profile with alanine transaminase expression greater than that of aspartate aminotransferase, which may be due to coinfection. However, initially in case 2, we observed mild transaminitis with an elevation of aspartate aminotransferase expression compared with that of alanine transaminase, which is consistent with dengue infection. Case 2 had leukopenia, which may be seen in both infections, which started to improve on day 3 of admission. In both cases, NS1 was negative, which could have been due to the late presentation. Treatment was mainly symptomatic. Intravenous fluid was avoided as COVID-19 patients are more prone to develop pulmonary edema [49]. Coinfections of two viruses can reduce or augment disease severity [50]. The most common outcome is viral interference, where one virus competitively suppresses the replication of the other [51]. This could have played an important role in changing the dynamics of dengue virus infection and the natural history of the disease. This could explain the two cases failing to demonstrate the typical features of dengue infection, both in its disease progression and the laboratory findings. More data are required to determine the changing dynamics of coinfection of the dengue virus with SARS-CoV-2. Furthermore, clinicians should use commercially available dengue serology judiciously with growing evidence of cross-reaction with other flaviviruses [52]. Limitations in this report include the inability to utilize the gold standard to diagnose dengue infection either by virus isolation or RT-PCR. In addition to this, dengue serotyping was also not done, which could have provided valuable information regarding virulence and disease dynamics.

## Conclusion

During this pandemic, it is important to consider other tropical diseases that are endemic to the tropics, such as dengue, Zika, chikungunya infections, or scrub typhus. Coinfection with two viruses may change the dynamics and natural history of disease progression, which may result in atypical presentation of dengue infection and COVID-19. In cases of COVID-19 with atypical presentations, such as increased hematocrit, depleted platelets, and transaminitis with a greater expression of aspartate aminotransferase than of alanine transaminase, dengue infection should be considered. In COVID-19 patients with positive dengue serology, it is important to repeat and reconfirm the test to avoid false positivity. It is important to keep an open mind when treating patients with COVID-19 and be vigilant of atypical signs and symptoms that may indicate any other tropical infection. Clinicians should be cautious in giving intravenous fluids when treating patients with COVID-19 and dengue coinfection, as COVID-19 patients are more prone to develop pulmonary edema.

## Data Availability

Not applicable.
